# Longitudinal Branched-Chain Amino Acids, Lifestyle Intervention, and Type 2 Diabetes in the Finnish Diabetes Prevention Study

**DOI:** 10.1210/clinem/dgac463

**Published:** 2022-08-02

**Authors:** Jemina Kivelä, Jelena Meinilä, Matti Uusitupa, Jaakko Tuomilehto, Jaana Lindström

**Affiliations:** Population Health Unit, Finnish Institute of Health and Welfare, 00271 Helsinki, Finland; Department of Public Health, University of Helsinki, 00014 Helsinki, Finland; Department of Food and Nutrition, University of Helsinki, 00014 Helsinki, Finland; Institute of Public Health and Clinical Nutrition, University of Eastern Finland, 70211 Kuopio, Finland; Population Health Unit, Finnish Institute of Health and Welfare, 00271 Helsinki, Finland; Department of Public Health, University of Helsinki, 00014 Helsinki, Finland; Saudi Diabetes Research Group, King Abdulaziz University, 80200 Jeddah, Saudi Arabia; Department of International Health, National School of Public Health, Instituto de Salud Carlos III, 28029 Madrid, Spain; Population Health Unit, Finnish Institute of Health and Welfare, 00271 Helsinki, Finland

**Keywords:** diabetes mellitus, type 2, amino acids, branched-chain, cluster analysis, lifestyle intervention, metabolome

## Abstract

**Context:**

Circulating branched-chain amino acids (BCAAs) are associated with the risk of type 2 diabetes (T2D).

**Objective:**

We examined to what extent lifestyle intervention aiming to prevent T2D interacts with this association and how BCAA concentrations change during the intervention.

**Methods:**

We computed trajectory clusters by k-means clustering of serum fasting BCAAs analyzed annually by mass spectrometry during a 4-year intervention. We investigated whether baseline BCAAs, BCAA trajectories, and BCAA change trajectories predicted T2D and whether BCAAs predicted T2D differently in the intervention (n = 198) and control group (n = 196).

**Results:**

Elevated baseline BCAAs predicted the incidence of T2D in the control group (hazard ratio [HR] 1.05 per 10 μmol/L, *P* = 0.01), but not in the intervention group. BCAA concentration decreased during the first year in the whole cohort (mean −14.9 μmol/L, *P* < 0.001), with no significant difference between the groups. We identified 5 BCAA trajectory clusters and 5 trajectory clusters for the change in BCAAs. Trajectories with high mean BCAA levels were associated with an increased HR for T2D compared with the trajectory with low BCAA levels (trajectory with highest vs lowest BCAA, HR 4.0; *P* = 0.01). A trajectory with increasing BCAA levels had a higher HR for T2D compared with decreasing trajectory in the intervention group only (HR 25.4, *P* < 0.001).

**Conclusion:**

Lifestyle intervention modified the association of the baseline BCAA concentration and BCAA trajectories with the incidence of T2D. Our study adds to the accumulating evidence on the mechanisms behind the effect of lifestyle changes on the risk of T2D.

Branched-chain amino acids (BCAAs), namely leucine, isoleucine, and valine, are essential amino acids that cannot be synthesized in the body, and they must therefore be derived from diet. Studies in different populations have repeatedly shown that elevated blood BCAA concentrations are associated with an increased risk of type 2 diabetes (T2D) (1-3), obesity (4), and insulin resistance (5). The concentration of BCAAs in the body is regulated by their degradation (6) and all BCAAs have similar degradation pathways in the body. Obesity and insulin resistance may partly diminish the degradation of BCAAs (7). At the cellular level, the association between circulating BCAA concentration and insulin resistance appears to be bi-directional; insulin resistance may increase BCAA concentration and high BCAA concentration may enhance insulin resistance (8).

Even though high circulating BCAA concentrations predict increased risk for T2D, the extent to which this association is independent of lifestyle factors is not clear. Lifestyle interventions promoting healthy diet with high intake of fruit and vegetables and whole grains, and restricted intake of high sugar and saturated fat combined with increased physical activity are known to decrease the risk for T2D, especially in high-risk people (9-13). Nevertheless, few studies have explored how lifestyle intervention may modify the association between circulating BCAAs and the risk of T2D or insulin resistance (14-17). These studies have been relatively small and lifestyle interventions and endpoints have varied, and therefore the results have remained inconsistent.

In the present study based on secondary analyses of Diabetes Prevention Study (DPS) participants, we explored how lifestyle intervention modifies the associations of baseline and longitudinal BCAAs with T2D incidence by creating trajectory clusters for BCAAs over a 4-year period.

## Methods

### Study Design and Participants

The Finnish Diabetes Prevention Study (NCT00518167, ClinicalTrials.gov) was a randomized controlled trial to investigate whether T2D is preventable by lifestyle intervention in individuals with high T2D risk. The active intervention was implemented between 1993 and 2001, and clinical follow-up lasted until 2009. The multicenter study comprised 522 overweight/obese (BMI > 25) individuals, aged 40-65 years and with mean 2-hour plasma glucose value of 2 oral glucose tolerance tests (OGTTs) indicating impaired glucose tolerance based on the World Health Organization (WHO) 1985 criteria (fasting plasma glucose < 7.8 mmol/L and plasma glucose 2 hours after glucose load 7.8-11.1 mmol/L) at baseline (18). The exclusion criteria included previous diabetes diagnosis, severe chronic diseases, and any diseases, conditions or drug treatments that may affect blood glucose values. The participants were randomly allocated either to the control group (n = 265) with general lifestyle advice or to the intervention group (n = 257) with intensive individual lifestyle counseling. During the follow-up period until 2009 there were 49 dropouts in the intervention group and 37 in the control group. The primary endpoint in the DPS was T2D diagnosis (19). The ethics committee of the National Public Health Institute in Helsinki (current Finnish Institute for Health and Welfare) Finland had approved the original study protocol and all participants gave written informed consent. For the postintervention follow-up, ethical approval was provided by the Northern Ostrobothnia Hospital District (PPSHP).

**Figure 1.**
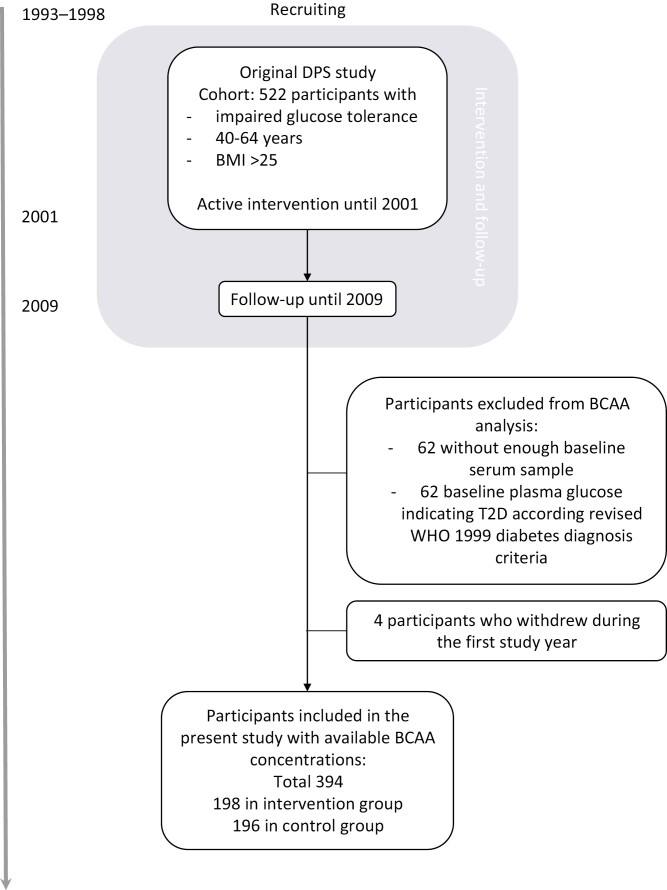
Flowchart of participants. Abbreviations: DPS, Diabetes Prevention Study; BMI, body mass index; BCAA, branched-chain amino acids; T2D, type 2 diabetes.

The study sample in the present secondary analysis comprises 126 men and 268 women who had measurements of BCAA concentrations available. The BCAA analysis was conducted in 2011 using frozen serum samples. The BCAA analysis was omitted due to lack of baseline serum sample (n = 62) and for people with baseline plasma glucose indicating T2D diagnosis according to the revised WHO 1999 diabetes diagnosis criteria (n = 62, fasting plasma glucose ≥ 7.0 mmol/L or plasma glucose 2 hours after glucose load ≥ 11.1 mmol/L) (20). In addition, we excluded from analysis the participants who withdrew from the study during the first study year (n = 4). The flowchart is presented in Fig. 1.

### Lifestyle Intervention

Goals of the intervention were 5% weight reduction, a diet with < 30% of daily energy intake from fat and < 10% from saturated fat, ≥ 15 g dietary fiber per 1000 kcal, and moderate physical activity at least 4 hours per week. The lifestyle counseling was conducted by a registered dietician or nutritionist mainly in individual sessions (7 times during the first year, every 3 months thereafter), complemented by some voluntary group sessions. Participants were individually encouraged to increase their physical activity and in addition, offered an opportunity to supervised exercise classes free of charge. Food intake and physical activity were monitored by food and exercise diaries. The participants included in the present study had a median intervention length of 4 years (range, 0-6 years). The details of the intervention and original study design are described in detail elsewhere (11, 19, 21, 22).

### Glucose Analyses and Type 2 Diabetes Diagnosis

During the annual study visits, a 2-hour OGTT was performed with a 75 g glucose load. Plasma glucose was determined in the local study center by standardized methods. T2D was diagnosed according to WHO 1985 criteria (18): fasting glucose ≥ 7.8 mmol/L or 2 hours after glucose load ≥ 11.1 mmol/L. The diagnosis was confirmed with a repeated OGTT if the first OGTT was indicating diabetes.

### BCAA Analyses

Serum samples were collected after an overnight fast. Samples were centrifuged and stored at −18 °C in the study clinics for up to the first 3 months and −70 °C thereafter until analyzed in 2011. Serum leucine, isoleucine, and valine concentrations were analyzed by mass spectrometry (TETHYS Bioscience, Inc., West Sacramento, CA). The serum samples were mixed with Deuterium-labeled standards, and the mixtures were derivatized with Tri-Sil and propyl chloroformate in n-propanol. The analytes were extracted into chloroform under nitrogen and reconstituted in iso-octane:chloroform. The 7890/5975 gas chromatography–mass spectrometry system (Agilent Technologies, CA) analyzed the solution with a ZB-50 column (Phenomenex, CA), using helium as the carrier gas. The single ion monitoring (SIM) mode with electron ionization was used for the mass spectrometric analysis, and the concentration was determined by comparing the peak to the relevant internal standard.

### Covariates

The covariates used in the statistical models were chosen based on the previous literature (17). The fully adjusted model covered clinical and lifestyle variables assumed to be associated with T2D or BCAA concentration. Model 1 was adjusted with age, sex, and study center (5 centers in Finland). Model 2 was additionally adjusted for the group allocation, education, smoking, baseline body mass index (BMI), baseline leisure-time physical activity, baseline saturated fat intake, baseline fiber intake, and baseline serum triglycerides. Participants reported their physical activity, education, and smoking status with a questionnaire. The study nurse measured weight and height with a coordinated protocol. Participants reported their food intake with 3-day food diaries using a picture booklet of portion sizes and package information to estimate the amounts of consumed foods. The diaries were recorded, and nutrient intakes calculated with the in-house Finessi program, based on Finnish Food Composition Database Fineli (23).

### Statistical Methods

The BCAA concentration was calculated as the sum of leucine, isoleucine, and valine concentrations. High and low baseline BCAA groups were created by categorizing the cohort by the median value. The change in BCAA concentration from baseline to year 1 was calculated by subtracting the baseline concentration from the concentration at the year 1 study visit.

The trajectory clusters of BCAA and BCAA change were composed to present the longitudinal changes in BCAA levels. The BCAA values from baseline to year 4 were used as the longitudinal data on 5 measure points with 1-year intervals. The change clusters (change from the baseline was calculated by subtracting the concentration at baseline) were composed to highlight the plausible changes in BCAA levels during the intervention study. Trajectories were computed by kml-package for R statistics (24, 25) which implements k-means to cluster longitudinal data. The participants with more than 2 missing values were excluded from the analysis, ending up with 363 participants. The Akaike Information Criterion (AIC) and the Bayesian Information Criterion (BIC) were used to find the best cluster model from computed models with 2 to 6 clusters (25). In addition, models with clusters having at least 5% of the population (≥ 20 participants) were considered valid to ensure reproducibility of the results. The model with fewest clusters was selected, considering AIC and BIC indexes and the clinical relevance of the solution.

The association of BCAAs and risk of incident T2D during the follow-up period was assessed by Cox proportional hazard regression models. The applied BCAA variables were baseline BCAAs as a continuous variable (µmol/L), change in BCAA concentration from the baseline to year 1 as continuous variable (µmol/L), trajectory clusters of BCAA levels from the baseline to 4 years, and trajectory clusters of change in BCAA levels from the baseline to 4 years. The follow-up time in Cox regression analyses was calculated from the enrollment date to the diagnosis of incident T2D or the end of the follow-up (year 2009) for participants without incident T2D. To test if the lifestyle intervention modified the association of BCAA and T2D risk, interaction terms of intervention allocation group and different BCAA variables were included in the Cox regression models. Analyses were conducted in the entire study population, and if the interaction term was significant, for the intervention and control groups separately.

We used mixed-effect regression model with random intercepts to examine a difference in the rate of change in BCAA concentration between the intervention and control groups. In addition, the number of control and intervention group participants was compared across trajectory clusters of BCAAs and BCAA change by Chi-squares.

A *P* value of < 0.05 was considered statistically significant. K-means clusters were analyzed with RStudio (26) with R language (27) and other analyses were performed with IBM SPSS Statistics for Windows version 25.0 (28).

## Results

### Characteristics

The characteristics of the study population are presented according to the intervention allocation in Table 1. The participants were middle-aged and overweight and 68% of them were women.

**Table 1.** Participant characteristics at baseline

	Intervention	Control
n	198	196
Women, %	66.2%	69.9%
BCAA, µmol/L	409.5 (70.5)	408.0 (71.2)
Age, years	55.5 (7.4)	55.4 (6.9)
Body mass index, kg/m^2^	31.1 (4.6)	31.1 (4.7)
Leisure-time physical activity, h/w	2.9 (3.5)	2.8 (3.1)
Saturated fat intake, E%	13.8 (3.5)	14.3 (3.6)
Energy intake, kcal/day	1774.6 (518.9)	1745.0 (517.7)
Fiber intake, g/1000 kcal	11.6 (4.0)	11.4 (3.8)
Blood triglycerides, mmol/L	1.7 (0.7)	1.7 (0.7)
Smoking, %	8.0%	12.0%
Higher education/academic degree, %	36.0%	32.0%
Study center		
Helsinki	23%	22%
Kuopio	17%	13%
Turku	21%	21%
Tampere	21%	21%
Oulu	18%	23%
HOMA-IR	3.8 (2.1)	3.8 (1.9)
Serum total cholesterol (mmol/L)	5.6 (1.0)	5.7 (0.9)

Values are mean (SD) or percentages.

Abbreviations: BCAA, branched-chain amino acids (sum of leucine, isoleucine, and valine concentrations); E%, proportion of total energy intake; HOMA-IR, homeostatic model of insulin resistance.

### Baseline BCAAs and Incidence of Type 2 Diabetes

**Figure 2.**
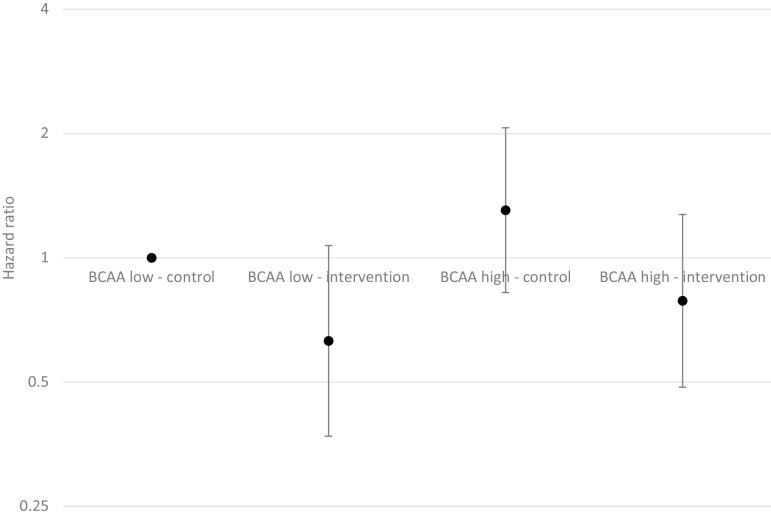
Joint effect of intervention and baseline branched-chain amino acids (BCAA) concentration on type 2 diabetes incidence. Participants in the control group with a low baseline BCAA comprise the reference group in Cox regression model adjusted for age, sex, education, smoking, body mass index (BMI), leisure-time physical activity, saturated fat intake, fiber intake, and blood triglycerides. The y-axis is on logarithmic scale.

In the entire cohort, participants with higher baseline BCAA concentrations had a higher hazard ratio (HR) for T2D during the follow-up period in model 1 (*P* = 0.002), but not in the fully adjusted model (Table 2). A statistically significant interaction (*P* = 0.005) between baseline BCAAs and intervention allocation for HR of T2D was detected, and the baseline BCAA concentration was associated with the T2D risk only in the control group (*P* = 0.01 in model 2). Comparing the HR for T2D in the intervention and control group participants by low and high baseline BCAA values (Fig. 2) demonstrates the interaction; the intervention group participants with a high baseline BCAA concentration had a similar HR for T2D with the control group participants with a low baseline BCAA concentration.

**Table 2.** Hazard ratio for type 2 diabetes according to the baseline BCAA concentration

	Overall		*P* value interaction	Intervention	*P* value	Control	*P* value
	HR (95% CI)	*P* value		HR (95% CI)		HR (95% CI)	
**Model 1**							
Per 10 µmol/L of BCAA	1.04 (1.01, 1.06)	0.002	<0.001	1.02 (0.98, 1.05)	0.37	1.06 (1.03, 1.09)	<0.001
**Model 2**							
Per 10 µmol/L of BCAA	1.02 (0.99, 1.05)	0.13	0.005	1.00 (0.96, 1.04)	0.84	1.05 (1.01, 1.09)	0.01

Model 1: adjusted for age, sex, and study center. Model 2: model 1 and additionally adjusted for group allocation, education, smoking and baseline body mass index (BMI), leisure-time physical activity, saturated fat intake, fiber intake, and blood triglycerides. Number of participants in the overall cohort in model 1 n = 394; model 2 n = 376; in intervention model 1 n = 198; model 2 n = 189; control model 1 n = 196; model 2 n = 187.

Abbreviations: BCAA, branched-chain amino acids (sum of leucine, isoleucine, and valine concentrations); HR, hazard ratio.

### Longitudinal Changes in BCAA Concentration and the Incidence of Type 2 Diabetes

**Figure 3.**
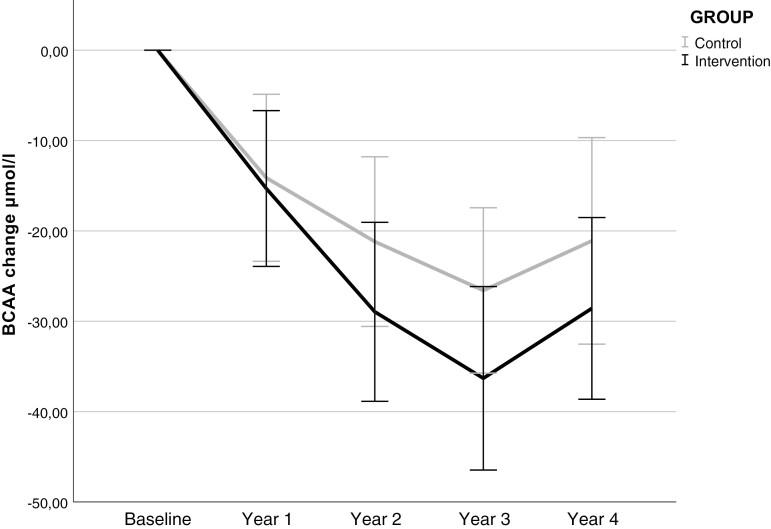
Branched-chain amino acid (BCAA) change from baseline to year 4 in the intervention and control groups. Error bars indicate 95% CI.

During the first year, the BCAA concentration decreased (−14.9 µmol/L [SD 58.5], *P* < 0.001) in the entire cohort. The change in BCAA concentration differed numerically but not statistically significantly between intervention and control groups (Fig. 3); at year 3 the change from baseline was −36.3 µmol/L [SD 66.9] in the intervention group and −26.6 µmol/L [SD 57.2] in the control group (difference for the groups *P* = 0.17). BCAA concentration slopes in time did not differ between the intervention and control groups (smaller decrease in control group, difference in slope 0.9 µmol per year, *P* = 0.54). The change in BCAAs from baseline to the year 1 visit was not associated with the risk of T2D (Table 3) in the entire study cohort and the interaction between the intervention allocation and BCAA change was not significant.

**Table 3.** Hazard ratio for type 2 diabetes according to the change in BCAA concentration from baseline to year 1

	Overall	*P* value	*P* value interaction	Intervention	p- value	Control	p- value
	HR (95% CI)			HR (95% CI)		HR (95% CI)	
**Model 1**							
Per 10 µmol/L BCAA change	1.03 (1.00, 1.07)	0.10	0.98	1.03 (0.98, 1.09)	0.24	1.02 (0.98, 1.07)	0.97
**Model 2**							
Per 10 µmol/L BCAA change	1.02 (0.98, 1.06)	0.34	0.06	1.03 (0.98, 1.09)	0.16	1.01 (0.96, 1.06)	0.79

Model 1: adjusted for baseline BCAA, age, sex, and study center. Model 2: model 1 and additionally adjusted for group allocation, education, smoking, baseline body mass index (BMI), leisure-time physical activity, saturated fat intake, fiber intake, and blood triglycerides. The number of participants in the overall cohort in model 1 n = 333, model 2 n = 317; in intervention group model 1 n = 164, model 2 n = 156; in control group model 1 n = 169, model 2 n = 161.

Abbreviations: BCAA, branched-chain amino acids (sum of leucine, isoleucine, and valine concentrations); HR, hazard ratio.

**Figure 4.**
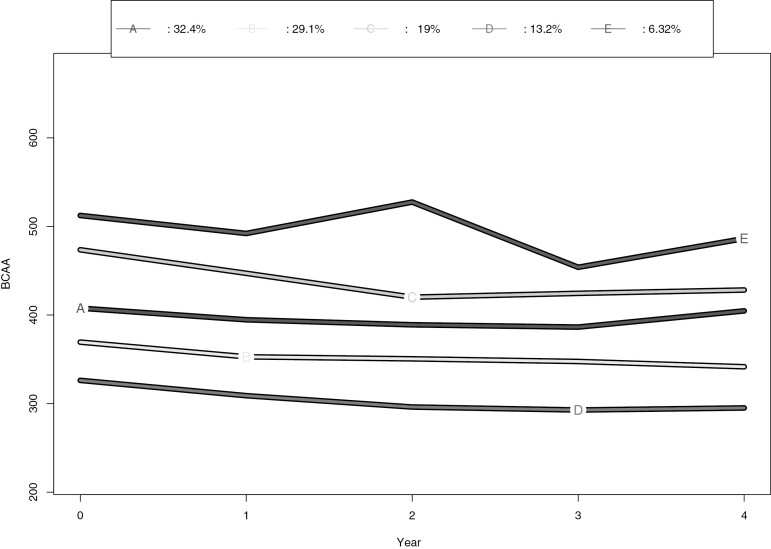
Trajectory clusters of BCAA levels from baseline to 4 years. Cluster size: A = 118, B = 106, C = 69, D = 48, E = 23.

The optimal clustering solution for BCAA trajectories identified 5 clusters of participants. The BCAA trajectories in clusters are presented in Fig. 4. The characteristics in the selected BCAA trajectory clusters are presented in Table 4. The trajectories started from different levels and most of them slightly decreased during the 4 years of follow-up. In the trajectory with highest mean (cluster E), the BCAA levels varied more over time than in the other trajectories.

**Table 4.** Participant characteristics in BCAA trajectory clusters

	A	B	C	D	E
n	118	106	69	48	23
Intervention, %	45.8%	51.9%	53.6%	43.8%	47.8%
Type 2 diabetes cases, n (%)*	49 (42%)	41 (39%)	31 (45%)	9 (19%)	13 (57%)
Women, %	66.9%	76.4%	56.5%	97.9%	21.7%
BCAA, µmol/L	407.8 (42.3)	369.4 (38.3)	473.7 (45.6)	326.2 (47)	512.4 (63.2)
BCAA first year change, µmol/L	-9.2 (59.6)	-13.5 (52.7)	-22.2 (68.5)	-17.3 (47.9)	-11.6 (74.1)
Age, years	55.7 (7)	56.1 (6.8)	55.1 (7.1)	54.8 (7.6)	54.2 (7.7)
Body mass index, kg/m^2^	31.6 (5)	30.3 (4)	31.4 (4.9)	30.1 (4.5)	31.4 (5.2)
Body mass index, first year change, kg/m^2^	-0.9 (1.7)	-1.1 (1.6)	-0.8 (1.6)	-2.1 (2.2)	-1.3 (1.8)
Leisure-time physical activity, hours/week	3.3 (4.0)	3.2 (3.4)	2.1 (2.3)	2.1 (2.7)	2.7 (2.2)
Saturated fat intake, E%	13.6 (3.1)	14.3 (3.8)	14.4 (3.5)	14.4 (4.1)	14.3 (4.1)
Fiber intake, g/1000 kcal	11.9 (3.9)	11.7 (3.7)	11.3 (3.7)	11.4 (4.9)	9.4 (2.6)
Blood triglycerides, mmol/L	1.7 (0.7)	1.4 (0.5)	1.8 (0.7)	1.1 (0.4)	1.8 (1.0)
HOMA-IR	3.8 (2.0)	3.2 (1.4)	4.5 (2.4)	2.8 (1.3)	5 (2.9)
HOMA-IR, first year change	-0.3 (1.6)	-0.3 (1.3)	-0.6 (3.3)	-0.3 (1.0)	-0.7 (2.4)
Higher education, %	35.6%	26.4%	34.8%	41.7%	39.1%
Smoking, %	5.1%	11.3%	10.1%	10.6%	4.3%

Values are at baseline if not stated otherwise. Values are mean (SD) or percentages.

Trajectory clusters (A-E) of BCAA comprised by longitudinal k-means: Cluster A quite stable trajectory with BCAA approx. 400 µmol/L; Cluster B slightly decreasing trajectory beginning at BCAA concentration approx. 370 µmol/L; Cluster C decreasing trajectory beginning at BCAA concentration approx. 470 µmol/L and ending at 430 µmol/L; Cluster D slightly decreasing trajectory starting at BCAA concentration approx. 320 µmol/L; Cluster E varying trajectory with approx. 510 µmol/L.

Abbreviations: BCAA, branched-chain amino acids (sum of leucine, isoleucine, and valine concentrations); E%, proportion of total energy intake; HOMA-IR, homeostatic model of insulin resistance; HR, hazard ratio.

*Number of type 2 diabetes cases ascertained by the end of 2009.

The HR for T2D was lowest in the cluster D with a low BCAA trajectory and highest in the cluster E with a high and varying BCAA trajectory (Table 5) in the entire cohort. A statistically significant interaction between the BCAA clusters and intervention allocation was detected for HR for T2D in model 1 (*P* = 0.01). Using cluster D as the reference (Table 5), the HR was significantly higher only for the cluster E in the intervention group (model 2, *P* = 0.03). In the control group, all other clusters had higher HR compared with the cluster D in model 1, but in the fully adjusted model only the HR for the clusters C vs D remained significant (*P* = 0.02). In the intervention group, the HR for the cluster C with decreasing trajectory was lower compared with the control group (HR 1.71 in intervention vs HR 4.21 in the control group in the model 2). The number of intervention and control participants in the clusters did not differ (*P* = 0.725).

**Table 5.** Hazard ratio for type 2 diabetes in BCAA trajectory clusters

	Overall	*P* value	Interaction *P* value *	Intervention	*P* value	Control	*P* value
	HR (95% CI)			HR (95% CI)		HR (95% CI)	
**Model 1**			0.01				
A vs D	2.89 (1.4, 5.96)	0.004		2.6 (0.96, 7.02)	0.06	3.14 (1.07, 9.26)	0.04
B vs D	2.60 (1.26, 5.38)	0.01		1.93 (0.69, 5.41)	0.21	3.30 (1.14, 9.57)	0.03
C vs D	3.35 (1.55, 7.25)	0.002		2.02 (0.67, 6.08)	0.21	5.42 (1.77, 16.53)	0.003
E vs D	5.87 (2.37, 14.49)	<0.001		4.8 (1.44, 16.03)	0.01	6.71 (1.65, 27.22)	0.01
**Model 2**			0.26				
A vs D	2.39 (1.06, 5.36)	0.03		2.52 (0.80, 7.93)	0.12	2.6 (0.78, 8.66)	0.12
B vs D	2.24 (1.01, 4.97)	0.05		2.28 (0.70, 7.44)	0.17	2.58 (0.83, 8.01)	0.10
C vs D	2.42 (1.04, 5.66)	0.04		1.71 (0.48, 6.08)	0.41	4.21 (1.23, 14.42)	0.02
E vs D	3.99 (1.46, 10.93)	0.01		4.53 (1.16, 17.79)	0.03	3.65 (0.76, 17.41)	0.11

Model 1: adjusted for age, sex, and study center. Model 2: model 1 and additionally adjusted for group allocation, education, smoking, body mass index (BMI), leisure-time physical activity, saturated fat intake, fiber intake, and blood triglycerides.

Trajectory clusters (A-E) of BCAA values comprised by longitudinal k-means: Cluster A quite stable trajectory with BCAA approx. 400 µmol/L; Cluster B slightly decreasing trajectory beginning at BCAA concentration approx. 370 µmol/L; Cluster C decreasing trajectory beginning at BCAA concentration approx. 470 µmol/L and ending at 430 µmol/L; Cluster D slightly decreasing trajectory starting at BCAA concentration approx. 320 µmol/L; Cluster E varying trajectory with approx. 510 µmol/L.

Number of participants in clusters, total/(intervention/control): A = 118 (54/64), B = 106 (55/51), C = 69 (37/32), D = 48 (27/21), E = 23 (12/11).

Abbreviations: BCAA, branched-chain amino acids (sum of leucine, isoleucine, and valine concentrations); HR, hazard ratio.

*Interaction *P* value; interaction for intervention allocation and trajectory clusters.

**Figure 5.**
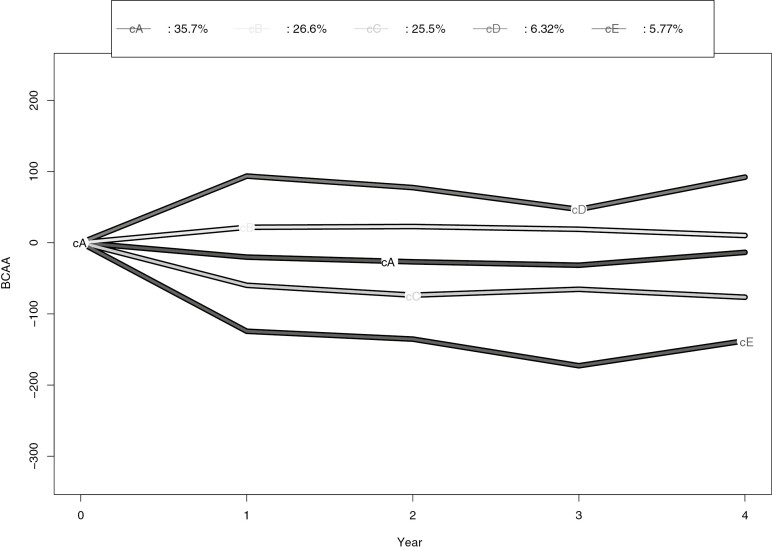
Trajectory clusters of branched-chain amino acid (BCAA) change. Cluster size: cA n = 130, cB n = 97, cC n = 93, cD n = 23, cE n = 21.

The optimal kml solution for BCAA change trajectories identified 5 clusters. The trajectories for the change in BCAAs from baseline in the clusters are presented in Fig. 5 and the characteristics of clusters in Table 6. The cluster with most participants (cluster cA) had a relatively stable trajectory. The trajectory cC decreased and the trajectory cE had even a stronger decrease with the follow-up time. The trajectory in cluster cB increased slightly and the trajectory cD increased to year 1, decreased to year 3, and increased again to year 4.

**Table 6.** Participant characteristics in BCAA change trajectory clusters

	cA	cB	cC	cD	cE
n	130	97	93	23	21
Intervention, %	51.5%	53.6%	47.3%	34.8%	66.7%
Type 2 diabetes cases, n (%)*	43 (33%)	34 (35%)	48 (52%)	9 (39%)	9 (43%)
Women, %	66.9%	63.9%	77.4%	69.6%	66.7%
BCAA, µmol/L	400.8 (53.9)	371.9 (57.1)	441.8 (55.6)	328.3 (47.1)	504.5 (77.3)
BCAA first year change, µmol/L	-20.2 (32.1)	21.7 (38.6)	-59.8 (35.0)	93.7 (47.0)	-124.4 (54.3)
Age, years	55.3 (7.3)	56.7 (6.6)	54.9 (7.0)	54.5 (7.5)	54.8 (7.7)
Body mass index, kg/m^2^	30.5 (4.0)	30.2 (4.0)	32.1 (5.4)	31.7 (6.0)	31.6 (4.9)
Body mass index change during first year, kg/m^2^	-1.2 (1.8)	-0.9 (1.3)	-1.3 (1.8)	-0.2 (1.7)	-1.9 (2.5)
Leisure-time physical activity, h/w	3.1 (3.3)	3.0 (3.8)	2.3 (2.6)	2.9 (3.3)	2.8 (4.1)
Saturated fat intake, E%	14.0 (3.8)	14.0 (3.5)	14.7 (3.6)	12.7 (3.2)	14.7 (2.4)
Fiber intake, g/1000kcal	11.2 (3.8)	12.2 (3.9)	11.5 (4.1)	10.9 (4.0)	10.7 (2.6)
Blood triglycerides, mmol/L	1.6 (0.7)	1.5 (0.6)	1.6 (0.7)	1.7 (0.9)	1.4 (0.5)
HOMA-IR	3.7 (2.1)	3.4 (1.7)	4.1 (2.3)	3.8 (1.8)	3.4 (1.4)
HOMA-IR change during first year	-0.6 (1.5)	0.0 (2.5)	-0.8 (2.0)	0.1 (1.3)	-0.4 (1.3)
Higher education, %	32.3%	33.0%	40.9%	13.0%	38.1%
Smoking, %	3.1%	9.3%	14.0%	17.4%	4.8%

Values are at baseline if not stated otherwise. Values are mean (SD) or percentages.

Trajectory clusters (cA-cE) of BCAA change from baseline to 4 years comprised by longitudinal k-means: Cluster cA quite stable trajectory; Cluster cB slightly increasing trajectory; Cluster cC slightly decreasing trajectory; Cluster cD increasing to first year then varying trajectory; Cluster cE decreasing trajectory.

Abbreviations: BCAA, branched-chain amino acids (sum of leucine, isoleucine, and valine concentrations); E%, proportion of total energy intake; HOMA-IR, homeostatic model of insulin resistance.

*Number of T2D cases ascertained by the end of 2009.

In the entire cohort, the HR for T2D was lower for the cluster cE with strongly decreasing trajectory than in the other clusters in model 1 (Table 7), but the HRs became smaller and nonsignificant in the fully adjusted model. A statistically significant interaction among the BCAA change clusters and intervention allocation was detected for HR for T2D incidence in the model 1 (*P* = 0.04). In the intervention group, the HR for clusters cC with slightly decreasing trajectory and cD with increasing trajectory were higher compared with the cluster cE in both models (model 2 *P* = 0.03 and *P* < 0.001, respectively). In the control group, the HR for T2D did not differ among the clusters. The number of intervention and control participants in the clusters did not differ (*P* = 0.261).

**Table 7.** Hazard ratio for type 2 diabetes in BCAA change trajectory clusters

	Overall	*P* value	Interaction *P* value *	Intervention	*P* value	Control	*P* value
	HR (95% CI)			HR (95% CI)		HR (95% CI)	
**Model 1**			0.04				
cA vs cE	2.05 (0.87, 4.85)	0.10		3.19 (0.92, 11.02)	0.07	1.60 (0.44, 5.73)	0.47
cB vs cE	2.64 (1.05, 6.64)	0.04		3.32 (0.87, 12.71)	0.08	2.64 (0.69, 10.17)	0.16
cC vs cE	2.68 (1.22, 5.89)	0.01		3.40 (1.05, 11.00)	0.04	2.41 (0.78, 7.45)	0.13
cD vs cE	4.00 (1.27, 12.58)	0.02		10.89 (1.59, 74.43)	0.01	2.70 (0.56, 13.07)	0.22
**Model 2**			0.55				
cA vs cE	1.73 (0.7, 4.29)	0.24		3.52 (0.88, 14.07)	0.08	1.28 (0.34, 4.86)	0.72
cB vs cE	2.24 (0.83, 6.06)	0.11		4.29 (0.95, 19.41)	0.06	2.06 (0.46, 9.33)	0.35
cC vs cE	2.00 (0.86, 4.67)	0.11		4.30 (1.13, 16.40)	0.03	1.7 (0.49, 5.84)	0.40
cD vs cE	2.95 (0.87, 10.04)	0.08		25.39 (2.83, 227.62)	<0.001	1.8 (0.32, 10.24)	0.51

Model 1: adjusted for age, sex, and study center. Model 2: model 1 and additionally adjusted for baseline BCAA concentration, group allocation (overall only), education, smoking, body mass index (BMI), leisure-time physical activity, saturated fat intake, fiber intake, and blood triglycerides.

Trajectory clusters (cA-cE) of BCAA change from baseline to 4 years comprised by longitudinal k-means: Cluster cA quite stable trajectory; Cluster cB slightly increasing trajectory; Cluster cC slightly decreasing trajectory; Cluster cD increasing to first year then varying trajectory; Cluster cE decreasing trajectory.

Number of participants in clusters, total/(intervention/control): cA = 130 (67/63), cB = 97 (52/45), cC = 93 (44/49), cD = 23 (8/15), cE = 21 (14/7).

Abbreviations: BCAA, branched-chain amino acids (sum of leucine, isoleucine, and valine concentrations); HR, hazard ratio.

*Interaction *P* value; *P* value for interaction of intervention allocation and trajectory clusters.

## Discussion

We found that a high baseline BCAA concentration predicted the risk of T2D, but this effect was seen only in the control group that was not given active lifestyle counseling. We showed that analyzing BCAA trajectories has a potential to improve prediction of T2D risk, especially when people are participating in a lifestyle intervention, and particularly BCAA change trajectories may provide additional information about the T2D risk. There was a statistically significant interaction for the intervention allocation and BCAAs, but no difference in changes of BCAA concentration after the first year or in the distribution of participants in the trajectory clusters between the intervention and control groups. Interestingly, the BCAA change trajectories were associated with the T2D risk in the intervention group only, suggesting that BCAA concentration changes predicted the incidence of T2D differently in the intervention and control groups. Our results are in line with our previous subsample analyses of the DPS study participants with BCAA concentration at year 1 (29).

We showed that BCAAs at baseline predicted T2D during a median of 11-year follow-up in a cohort of middle-aged men and women with impaired glucose tolerance, but this association was statistically significant only in the group without active lifestyle counseling. In the intervention group, the association probably diminished because the participants engaged in the lifestyle changes which changed the individuals’ BCAA concentration and the risk for T2D. The association between BCAAs and T2D has been shown in different populations (2, 30, 31), and BCAAs have predicted T2D up to 19 years before the diagnosis of T2D (2). Our results in the control group are in line with a meta-analysis of 8000 individuals from 8 studies, which revealed a 36% higher risk for T2D per 1 SD of baseline BCAA (5).

To our knowledge, our study is the first to explore the association of BCAA and T2D in trajectory models in a lifestyle intervention setting. In previous studies, longitudinal associations between BCAAs and insulin resistance or T2D have been explored with 2 measuring points. In a Mediterranean diet intervention (17) on participants with cardiovascular risk factors (n = 892) an increase in BCAAs from baseline to year 1 was associated with an increased T2D risk in control group, but not in the intervention groups. In a lifestyle intervention study with 266 adults (15), a decrease in BCAAs in the intervention group was not associated with the change in analyzed glycemia measures after a 2-year follow-up. In adolescents (n = 33) the BCAA concentration was associated with insulin resistance after a 6-month lifestyle intervention (14), but the change in BCAA concentration during the intervention was not significant and no association between the changes in BCAAs and changes in glycemia measures from baseline was seen. A 7-day BCAA-restricted diet (16) decreased BCAA concentration and reduced insulin resistance in healthy volunteers (n = 12). These studies suggest that there might be an interaction between BCAA and lifestyle intervention for the T2D risk or insulin resistance. Our study adds to the evidence with a long follow-up, reliable T2D detection and having BCAA measures in more than 2 time points.

The increase in BCAA from baseline to year 1 was associated with a higher T2D risk in a Mediterranean diet intervention study (17), but in our study we did not see such association. The lack of association might indicate that the level of BCAAs is more important than the change in BCAA concentration. Additionally, there might be too much random intra-individual variation for simple change from one point to another to model the change precisely. To model statistically the BCAA level and its change simultaneously in multiple time points, we implemented the trajectory cluster model to the data. In our study population, the risk for T2D in the trajectory clusters increased according to the higher mean BCAA levels in the trajectories. In the intervention group, the risk for T2D in the trajectory cluster with the second-highest but downward BCAA levels (cluster C) did not differ from the cluster with the lowest BCAA levels (cluster D), but in the control group, the risk for T2D was 5-fold in the second-highest cluster compared with the lowest. This finding suggests that downward BCAA trajectory was a marker of decreasing risk for T2D in the intervention but not in the control group. The risk for T2D in the cluster with high and varying BCAA levels (trajectory E) was particularly high in the intervention group. One reason could be that participants in the cluster E experienced relapses in lifestyle maintenance, leading to BCAA cycling. BCAA cycling might indicate weight cycling which has been associated with higher T2D risk in another study (32).

To highlight the changes in BCAA levels, we computed change trajectories in addition to the trajectories of BCAA levels. Interestingly, the difference in the risk for T2D between upward trajectory (cluster cD) and downward trajectory (cluster cE) was seen only in the intervention group. This finding indicates that the risk for T2D may depend on the reason why BCAA levels changed; if BCAA levels decrease because of healthy lifestyle changes, the decrease in BCAA levels seems to have a stronger impact on T2D risk. In a Mediterranean diet intervention study of Ruiz-Canela et al (17), the BCAA change was associated with T2D in the control group only, but in our trajectory models we showed the change trajectories predicted T2D only in the intervention group. Our conclusion is somewhat opposite to Ruiz-Canela et al (17), who indicated the dietary intervention attenuated the direct association between BCAAs and the T2D risk since we showed significant association with the T2D risk only in the intervention group. A longer follow-up time in our BCAA trajectory models and differences in intervention diets might explain part of the discrepancy in results between the 2 studies and their conclusions.

In our study, the BCAA concentration decreased in the entire study population, but there was no difference in the change of BCAA concentrations between the intervention and the control groups during the first year. The distribution of intervention and control participants in the BCAA clusters did not differ either. Previous studies have detected that different lifestyle interventions (14, 15, 17) and weight-loss diets (33) have led to a decreased circulating BCAA concentration after a one-year follow-up. The absence of difference between the intervention and control group in our study might have been due to differences in the lifestyle intervention or lack of power. Furthermore, while a 4.5 kg weight reduction was seen in the intervention group, the control group participants also made changes in their lifestyle and lost on average 0.8 kg weight during the first year (22). The lack of “true” control group without any kind of intervention might have diminished the difference between the groups.

Recent studies demonstrate that the physiological mechanisms linking increased BCAA with obesity and T2D are subject of an active research topic (8). Gene variants (34), metabolism in white adipose tissue and liver cells (35), the gut microbiome (36), lipid accumulation in muscle cells (37), and decreased tryptophan metabolism in the nervous system (38) are some examples of suggested mechanisms linking BCAA overload with T2D.

For the first time, we have presented the BCAAs as trajectories over 4 years and studied how the lifestyle intervention of the DPS modified the association of BCAAs and the T2D risk. The lifestyle intervention in the DPS has been a model for many later successful interventions to prevent T2D (39). During the DPS intervention, all participants were followed with yearly OGTTs regularly, which is exceptional in a study with hundreds of participants. The participants were followed up to 15 years, which is one of the longest follow-ups in such studies. As a limitation to the generalizability of our results, all participants were middle-aged, obese, had impaired glucose tolerance, and presented white Caucasian ethnicity. Since in some analyses, the associations were not very strong, a bigger population might have revealed more robust results. The population in this substudy is not directly comparable with the populations presented in the previous DPS publications because the BCAAs were analyzed only for those people who did not have diabetes according to the revised WHO diabetes diagnosis criteria, and because of the missing blood samples. Nevertheless, the excluded participants were evenly distributed among the intervention and control groups and therefore the groups remained comparable to each other. We used Kml, a k-means clustering method to find clusters in the longitudinal data. K-means is a nondeterministic method, which considers the distance between the measured variables and suits smaller sample sizes and for trajectories with changing slopes. K-means clustering is ideal for exploratory settings, but it is not possible to test how well the model agrees with a theoretical model or with another dataset.

Our results illustrated that the DPS lifestyle intervention modified the association between serum BCAAs and T2D risk in people with a high risk for T2D. Changes in circulating BCAA levels predicted T2D risk, especially with people participating in the lifestyle intervention. Our study adds to the accumulating evidence on the mechanisms behind the effect of lifestyle changes on the risk of T2D.

## Data Availability

Restrictions apply to the availability of some or all data generated or analyzed during this study to preserve patient confidentiality or because they were used under license. The corresponding author will on request detail the restrictions and any conditions under which access to some data may be provided.

## Abbreviations

BCAA: branched-chain amino acid
BMI: body mass index
DPS: Diabetes Prevention Study
HR: hazard ratio
OGTT: oral glucose tolerance test
T2D: type 2 diabetes
WHO: World Health Organization

## Notes

Grants or fellowships supporting the writing of the present article: Päivikki and Sakari Sohlberg Foundation; Yrjö Jahnsson Foundation; Juho Vainio Foundation; the Academy of Finland, under the project “Data-driven solutions to improve prevention of type 2 diabetes” (grant 332466).

## Clinical Trial Information

ClinicalTrials.gov registration number: NCT00518167.
